# Supplementary material for: PlatyphyllenoneExerts Anti-Metastatic Effects on Human Oral Cancer Cells by Modulating Cathepsin L Expression, MAPK Pathway and Epithelial–Mesenchymal Transition
Source: Int J Mol Sci. 2021 May 9;22(9):5012. doi: 10.3390/ijms22095012 (PMC8125947; doi:10.3390/ijms22095012)
Supplement: Supplementary file 1 [file ijms-22-05012-s001.zip › ijms-1211495-supplementary.pdf]

Supplementary figure 1

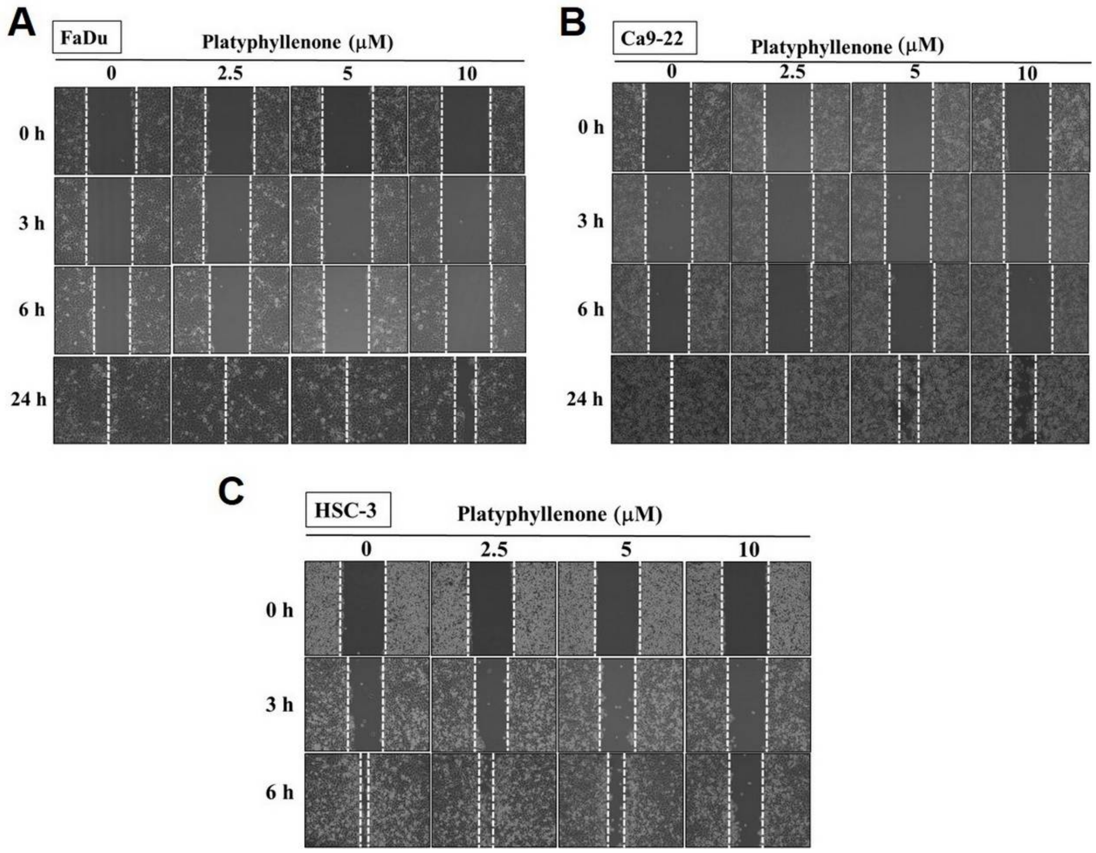

Supplementary figure S1. Platyphyllenone inhibits motility of human oral cancer cells. Wound closer assay was conducted to assess the human oral cancer cell motility in platyphyllenone-treated (A) FaDu, (B) Ca9-22 and (C) HSC3 cells.

Supplementary figure 2

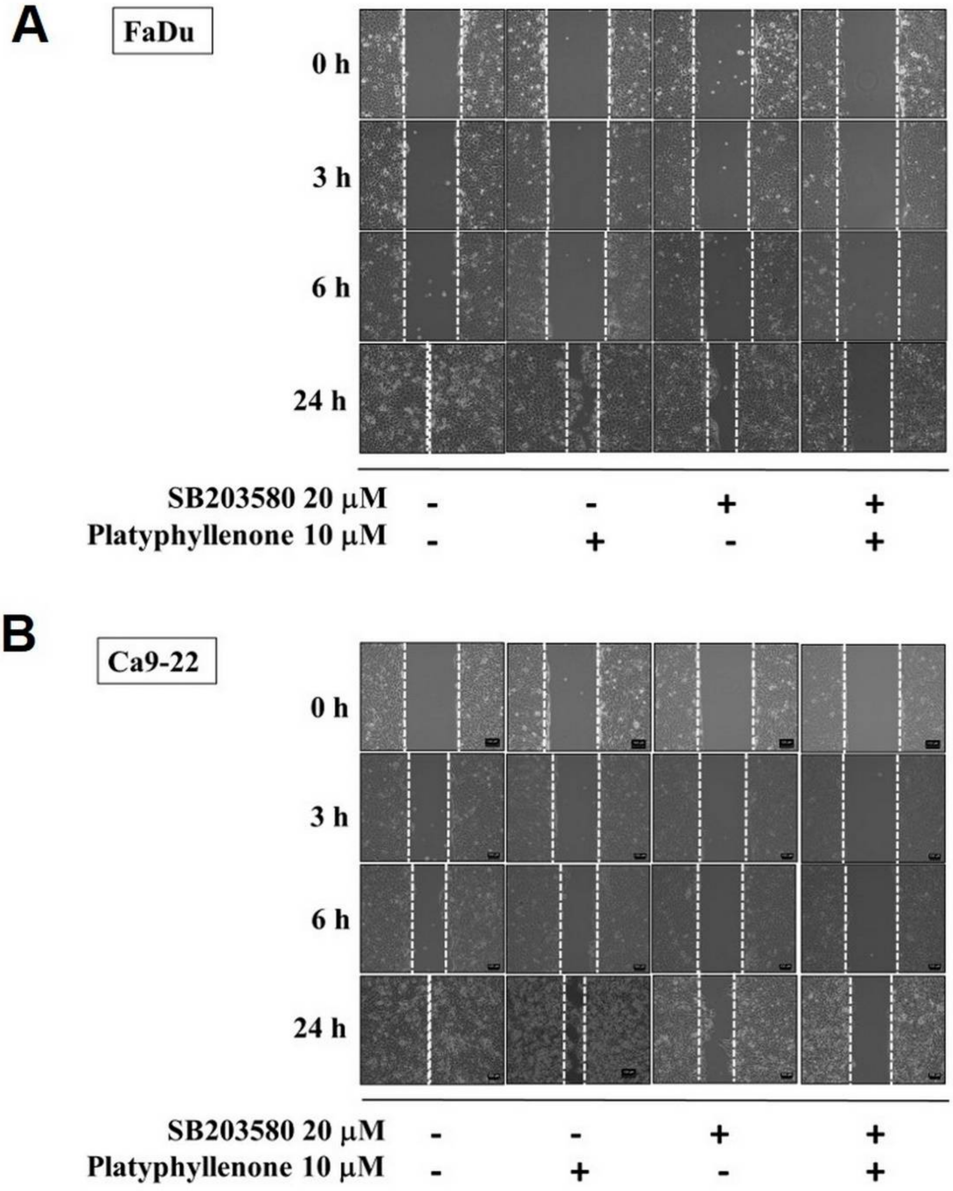

Supplementary figure S2. Effect of platyphyllenone and SB203580 co-treatment on oral cancer cell motility. Wound closer assay was conducted to assess the cell motility in platyphyllenone and SB203580-cotreated FaDu (A) and Ca9-22 cells (B).
